# A highly efficient and sustainable photoabsorber in solar-driven seawater desalination and wastewater purification†

**DOI:** 10.1039/d3ra01938a

**Published:** 2023-06-14

**Authors:** Masoomeh Shafaee, Elaheh K. Goharshadi, Mohammad Mustafa Ghafurian, Mojtaba Mohammadi, Hassan Behnejad

**Affiliations:** a Department of Physical Chemistry, School of Chemistry, University College of Science, University of Tehran Tehran 14155 Iran h.behnejad@ut.ac.ir; b Department of Chemistry, Faculty of Science, Ferdowsi University of Mashhad Mashhad Iran gohari@um.ac.ir +98 9177948974; c Nano Research Centre, Ferdowsi University of Mashhad Mashhad Iran; d Center for Nanotechnology in Renewable Energies, Ferdowsi University of Mashhad Mashhad Iran; e Mechanical Engineering Department, Ferdowsi University of Mashhad Mashhad Iran; f Department of Physics, Faculty of Science, Ferdowsi University of Mashhad Mashhad Iran

## Abstract

Producing freshwater from seawater and wastewater is of great importance through interfacial solar steam generation (ISSG). Herein, the three-dimensional (3D) carbonized pine cone, CPC1, was fabricated *via* a one-step carbonization process as a low-cost, robust, efficient, and scalable photoabsorber for the ISSG of seawater as well as a sorbent/photocatalyst for use in wastewater purification. Taking advantage of the large solar-light-harvesting ability of CPC1 due to the presence of carbon black layers on the 3D structure, its inherent porosity, rapid water transportation, large water/air interface, and low thermal conductivity, a conversion efficiency of 99.8% and evaporation flux of 1.65 kg m^−2^ h^−1^ under 1 sun (kW m^−2^) illumination were achieved. After carbonization of the pine cone, its surface becomes black and rough, which leads to an increase in its light absorption in the UV-Vis-NIR region. The photothermal conversion efficiency and evaporation flux of CPC1 did not change significantly during 10 evaporation–condensation cycles. CPC1 exhibited good stability under corrosive conditions without significant change in its evaporation flux. More importantly, CPC1 can be used to purify seawater or wastewater by the removal of organic dyes as well as by the reduction of polluting ions, like nitrate ions in sewage.

## Abstract 2

Producing freshwater from seawater and wastewater is of great importance through interfacial solar steam generation (ISSG).

## Graphical abstract

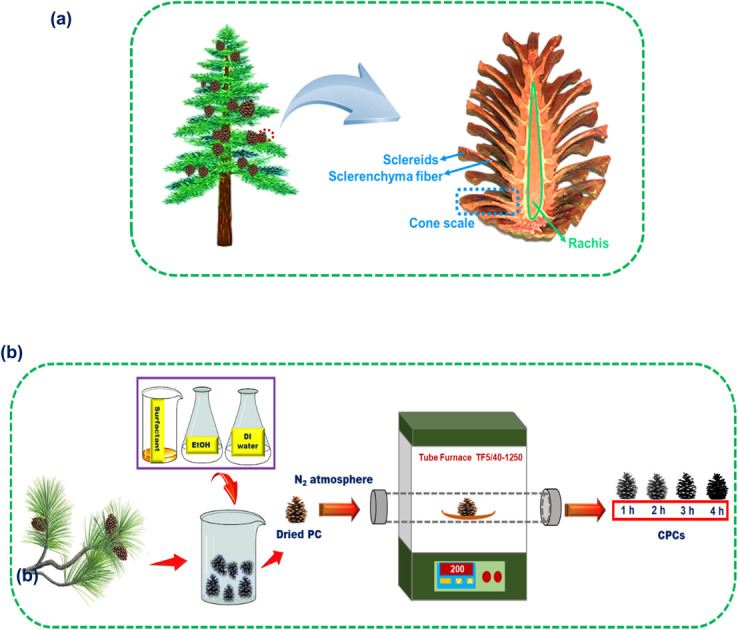

## 1. Introduction

Nowadays, the fast development of industrialization, population growth, and climate change are playing a role in the increasing pollution of water resources.^1–3^ Moreover, there is a severe shortage of drinking water resources in the world.^4,5^ Hence, the supply of freshwater using salt water desalination or wastewater treatment is very necessary and vital. Traditional freshwater production methods, such as reverse osmosis,^6–8^ multi-stage flash distillation,^9^ multi-effect distillation,^10^ electrodialysis/electrodialysis reversal,^11^ membrane distillation,^12,13^ and capacitive deionization^14,15^ are facing three key challenges: complex equipment requirements, high energy consumption, and high cost.^16,17^ To address these challenges, green, and eco-friendly interfacial solar steam generation (ISSG) from seawater, brackish water, and sewage has been developed as a method to produce freshwater *via* photothermal evaporation using green and sustainable solar energy.^18–25^ By utilizing solar energy, heat is localized at the water/air interface and hence heat loss is minimized in the ISSG technique.^26,27^ An ISSG device or photoabsorber is usually composed of a photothermal material and a substrate. A photothermal material absorbs solar photons and converts them to thermal energy.^28^ To date, several photothermal materials, including metallic nanoparticles (NPs) (Au NPs^29^ and Cu NPs^30^), metal oxide NPs (TiO_2_,^31^ VO_2_,^32^ tungsten oxide (WO_*x*_),^33^ and MnO_2_ (ref. 34)), zero-dimensional (0D) to three-dimensional (3D) carbon-based materials (reduced graphene oxide^35^ and graphitic carbon nitride^36^), and polymers^37^ have been used. A suitable substrate for use in ISSG should transfer water continuously to its hot surface. A substrate should be hydrophilic and porous in order to transport water to its hot surface. Also, it should have low thermal conductivity (TC) to minimize heat loss to the bulk. Several substrates, including carbon foam, polystyrene foam, aerogels, polymeric compounds, and wood, have been used in ISSG systems.^38–44^ In some ISSG devices, a versatile photoabsorber that can act both as a photothermal material and as a substrate is used.^45,46^ Although several photoabsorbers have been designed for use in ISSG systems, several challenges, including the cost and toxicity of raw materials, biocompatibility, complexity of the preparation process, and stability, still remain.^34,47,48^ Designing an efficient photoabsorber is of great importance to overcome these challenges. A photoabsorber with a 3D structure can increase the overall evaporation surface and reduce the enthalpy of vaporization *via* recycling latent heat released from the vapor condensation. Moreover, 3D structures need to harvest additional energy from the surrounding air, convective flow, and bulk water to optimize the evaporation performance of a photoabsorber.^49–51^ In recent years, highly efficient, porous, and cost-effective 3D photoabsorbers based on natural materials have been designed. Xu *et al.*^49^ carbonized mushroom and used it as a photoabsorber in ISSG. The heat loss to the environment was reduced by the mushroom geometry. Fang *et al.*^52^ achieved a conversion efficiency of 86.5% under 1 sun by using porous carbonized lotus seedpods. Water was absorbed through the hierarchical meso–macroporous structure forming interconnected porous networks leading up to the surface for steam generation. The heat loss was minimized by the unique macroscopic cone shape of the carbonized lotus. Long *et al.*^53^ achieved a conversion efficiency of 127.8% under 1 sun using the pyrolysis of ethanol-treated carrot. The lignin and β-carotene were removed to create the internal channels in the carrot structure. The microchannels provided water continuously to the surface and heat loss was avoided. Sun *et al.*^50^ used a carbonized sunflower head as a photoabsorber and achieved an evaporation efficiency and flux of 100.4% and 1.51 kg m^−2^ h^−1^, respectively, under 1 sun. Liu *et al.*^54^ prepared two layers of an efficient photoabsorber using a loofah sponge. The top layer was carbonized for light absorption and the bottom layer was concerned with sufficient water transportation. An evaporation efficiency and flux of 89.9% and 1.42 kg m^−2^ h^−1^ were recorded under 1 sun, respectively. Chen *et al.*^55^ fabricated an anisotropic porous framework carbonized-corncob, a kind of agriculture waste, to reduce thermal energy loss and to enhance light trapping, in which the evaporation efficiency and flux were measured as 86.7% and 1.36 kg m^−2^ h^−1^ under 1 sun, respectively. Feng *et al.*^56^ prepared a photoabsorber using a torrefaction bamboo with an evaporation efficiency and flux of 94% and 1.522 kg m^−2^ h^−1^ under 1 sun, respectively.

**Fig. 1:**
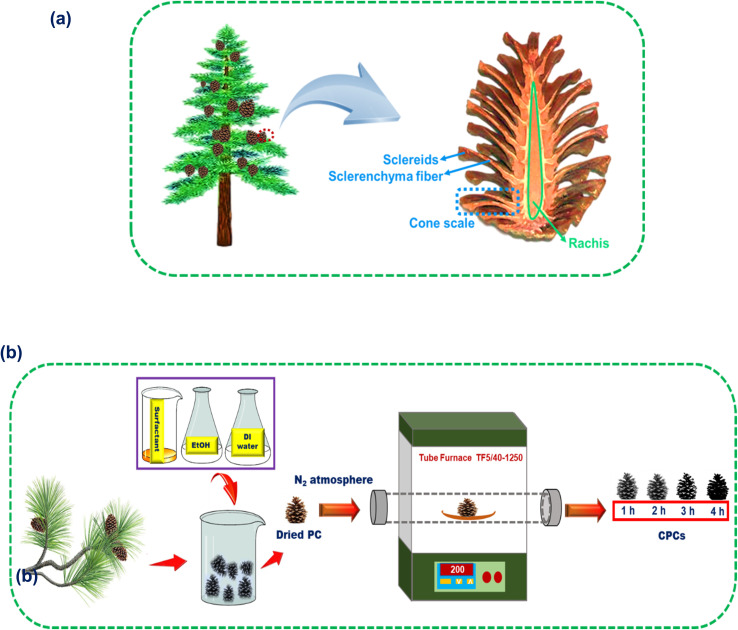
(a) Representation of the inner structure of a pine cone in a longitudinal section. (b) Schematic illustration of the preparation of the CPCs.

Pine cone (PC) displays a hygroscopic behavior in nature. Under dry circumstances, the pine cone opens for broadcast sowing by wind, while the closed form of the pine cone is a good strategy used to protect the seeds on rainy days.^57^ The pine cone (*Pinus eldarica*) in this work was composed of cone scales arranged in an 8, 13 Fibonacci sequence around a central rachis with vertically arrayed channels for water pumping.^58^ The 3D helical arrangement of the cone scales creates a structure for enhancing the water/air interface area, which is beneficial for steam escaping and for trapping solar light from different incident angles to prevent its reflection for efficient water evaporation. A cone scale includes a bilayer structure of sclerenchyma fibers and sclereids. Fig. 1a illustrates the inner structure of the pine cone in a longitudinal section. The cone scales react to humidity change through different orientations of the sclerenchyma fibers ratio to the longitudinal axis, which lead to shrinking and swelling movements. This change of cone scales is required for absorbing a little amount of water.^57,58^ Moreover, the continuous transport of water to the surfaces of the scales is performed *via* the hierarchical porous structure of the PC. PC is a plentiful and accessible biowaste resource in most parts of the world as a lignocellulosic material. The inherent hydrophilicity, appropriate thermal and mechanical properties,^59^ and high sunlight trapping capability are some merits that have seen the PC tested for several applications as a biosorbent^60^ and solar steam generator.^61,62^

There are some reports in the literature about the use of pine cone-based photoabsorbers modified by a polymer^61^ and molybdenum disulfide.^62^ Herein, instead of modifying pine cone by expensive/toxic chemicals, it was carbonized and used as a low-cost, highly efficient, and versatile biocompatible photoabsorber in the ISSG of seawater as well as in wastewater purification. Besides, carbonized pine cone (CPC) can decrease the concentration of ions in paper industry sewage. The porous structure and rough surface during carbonization achieved by morphology manipulation of the pores of the pine cone can ensure adequate water transport channels. The absorbance of solar-light energy is increased after carbonization while the porosity of the pine cone prevents the accumulation of salt fouling. CPC was used here to purify seawater as well as real wastewater. The desalination performance of CPC was assessed systematically under normal and harsh conditions. Also, CPC was used for the removal of a dye.

## 2. Materials and methods

### 2.1. Materials

Ethanol (EtOH, 96%) was obtained from Mojallali Co. and nitrogen gas (99.99%) from Arian Co. Methyl orange (MO) dye was purchased from Merck. The pine cones were collected from the campus of Ferdowsi University of Mashhad. Caspian seawater and wastewater samples from Part Papyrus Paper Industries (Fariman, Razavi Khorasan province, Iran) were used as the sources of brackish water.

### 2.2. Instruments

An ultrasonic bath (DSA 100-SK2-4.0 L) was used for washing the pine cones. The oven SH2007 (PAAT-ARIYA Co) was used to dry the samples. The pine cones were carbonized with a furnace TF5/40-1250 (AZAR FURNACES Co) under nitrogen gas. The X-ray diffraction (XRD) patterns of the samples were recorded using a Bruker/D8 Advanced diffractometer in the 2*θ* range from 12° to 60°, in 0.04° steps using Cu Kα (*λ* = 0.15406 nm) radiation. The UV-Vis-NIR absorbance spectra of the samples were obtained using an Avantes-ULS2048 spectrophotometer. The FTIR spectra of the samples were obtained using a KBr pellet on a Thermo Nicolet-Avatar 370 spectrometer at room temperature. The wettability of the samples was measured using a contact angle analyzer system (5V-USB port power source) from Adecco Company with a 0.1° accuracy and including a CCD camera and IrcA96 software. Thermogravimetric analysis (TGA) of PC was performed using a Thermal Analysis-TA (TGA Q50) instrument from room temperature to 600 °C at a heating rate of 20 °C min^−1^ under a nitrogen atmosphere. The morphology of the surface and cross-section of the pine cones were determined by scanning electron microscopy (SEM) (VP 1450, LEO). A solar simulator system with a 750 W Xe lamp (SS301) from NTN Arvin Co. was used for the ISSG experiments. The mass changes of seawater during the tests were recorded using an electronic balance with a 0.01 g accuracy from Kern Co. To record the temperature, three temperature sensors (Model LM35AH) with 0.01 °C accuracy, at the top, medium, and bottom, were used which were placed at distances of 1, 2 and 3 cm from the container, respectively. The transmission of the temperature data to the computer was performed by a data logger (eak-452, Electro Adin Khavaran Co).

The TC of the samples was measured using KD2 pro apparatus with an SH.1 sensor. The thermal images were taken using an FLIR ONE thermal camera (P/N 435-0003-01-00). The ion concentrations, electrical conductivity, and pH of the seawater or wastewater before and after solar desalination were measured by inductively coupled plasma mass spectrometry (ICP-MS) on a 76004550 model instrument from Spectro Arcos Company, Electro conductometer-644 device (Switzerland Metrohm Company), and a pH meter (Metrohm ion analysis model, Switzerland), respectively. The UV-vis absorption spectra of the samples were recorded using a UVD-2950 spectrophotometer.

### 2.3. Preparation of the carbonized pine cones

At first, the pine cones were washed by a surfactant, aqueous solution of EtOH, and deionized (DI) water in an ultrasonic bath for 30 min to remove any impurities. Then, the pine cones were dried in the oven for 24 h at 50 °C. The dried pine cones were carbonized in a furnace under nitrogen gas at 200 °C with a heating rate of 5 °C min^−1^ for 0, 1, 2, 3, and 4 h and the samples were named as PC, CPC1, CPC2, CPC3, and CPC4, respectively. Fig. 1b shows a schematic illustration of the preparation of the CPCs.

### 2.4. ISSG experiments

**Fig. 2:**
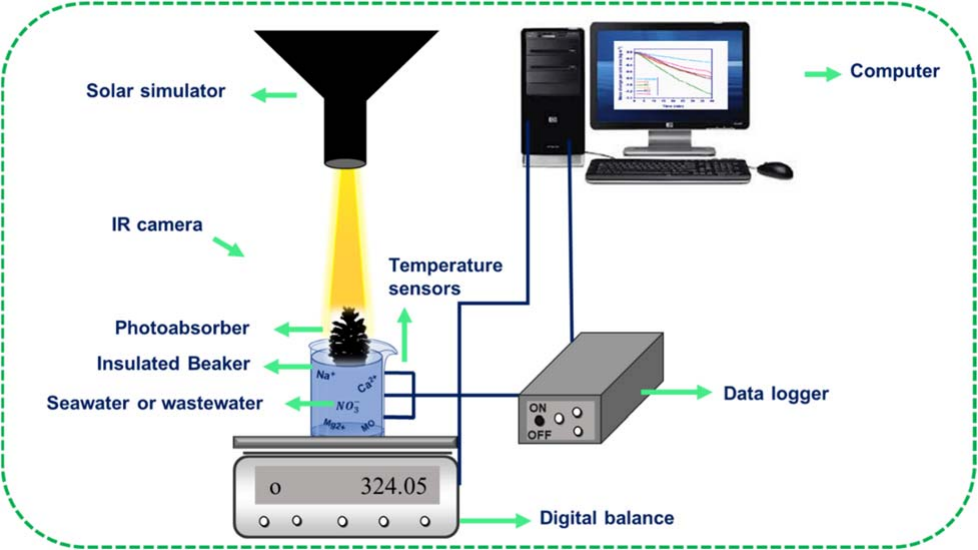
Schematic representation of the ISSG setup.

The prepared samples, namely PC, CPC1, CPC2, CPC3, and CPC4, were used as photoabsorbers. The ISSG experiments were conducted at a temperature between 29 °C and 32 °C and humidity of ∼46% using a solar simulator system. To suppress the thermal dissipation of the bulk seawater or wastewater to the surrounding, the solar receiver (glass chamber) was formed of an insulated glass beaker with 100 mL of Caspian seawater or wastewater. Then, the photoabsorber was put on the surface of the bulk water. The photoabsorber was surrounded by a polyethylene foam, both as a thermal insulator with a TC of 0.214 W m^−1^ K^−1^ (ref. 63) and as a holder on the water surface. Besides, three sensors and an infrared thermal camera were used to record the temperatures of the bulk seawater and the surface of the photoabsorbers, respectively. A schematic representation of the ISSG setup is shown in Fig. 2. The evaporated mass change over time was monitored by an electronic balance under dark conditions and simulated sunlight for 40 min to calculate the photo-to-thermal conversion efficiency, *ƞ*, according to eqn (1):^64^1
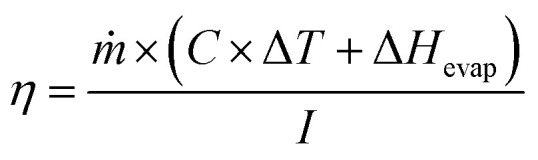
where *ṁ* is the seawater mass change per unit area per solar irradiation time, *C* is the specific heat capacity of the seawater (3.85 kJ kg^−1^ °C^−1^), Δ*T* is the average temperature difference of the bulk seawater after and before solar irradiation, Δ*H* is the evaporation enthalpy of pure water (2257 kJ kg^−1^) at 1 atmosphere, and *I* is the power density of solar illumination in terms of sun. The solar power density as a function of the height of the best photoabsorber was measured and is shown in Fig. S1.†

The seawater evaporation flux, *ṁ*, was according to eqn (2):^24^2
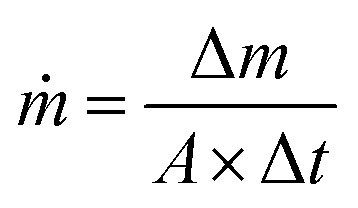
where Δ*m*, *A*, and Δ*t* are the seawater mass change in the presence of simulated sunlight subtracted from that in the dark, the effective surface area of a photoabsorber, and the solar irradiation time (40 min), respectively. The mass change per unit area of the best photoabsorber under the dark condition is shown in Fig. S2.†

## 3. Results and discussion

### 3.1. Characterization

**Fig. 3:**
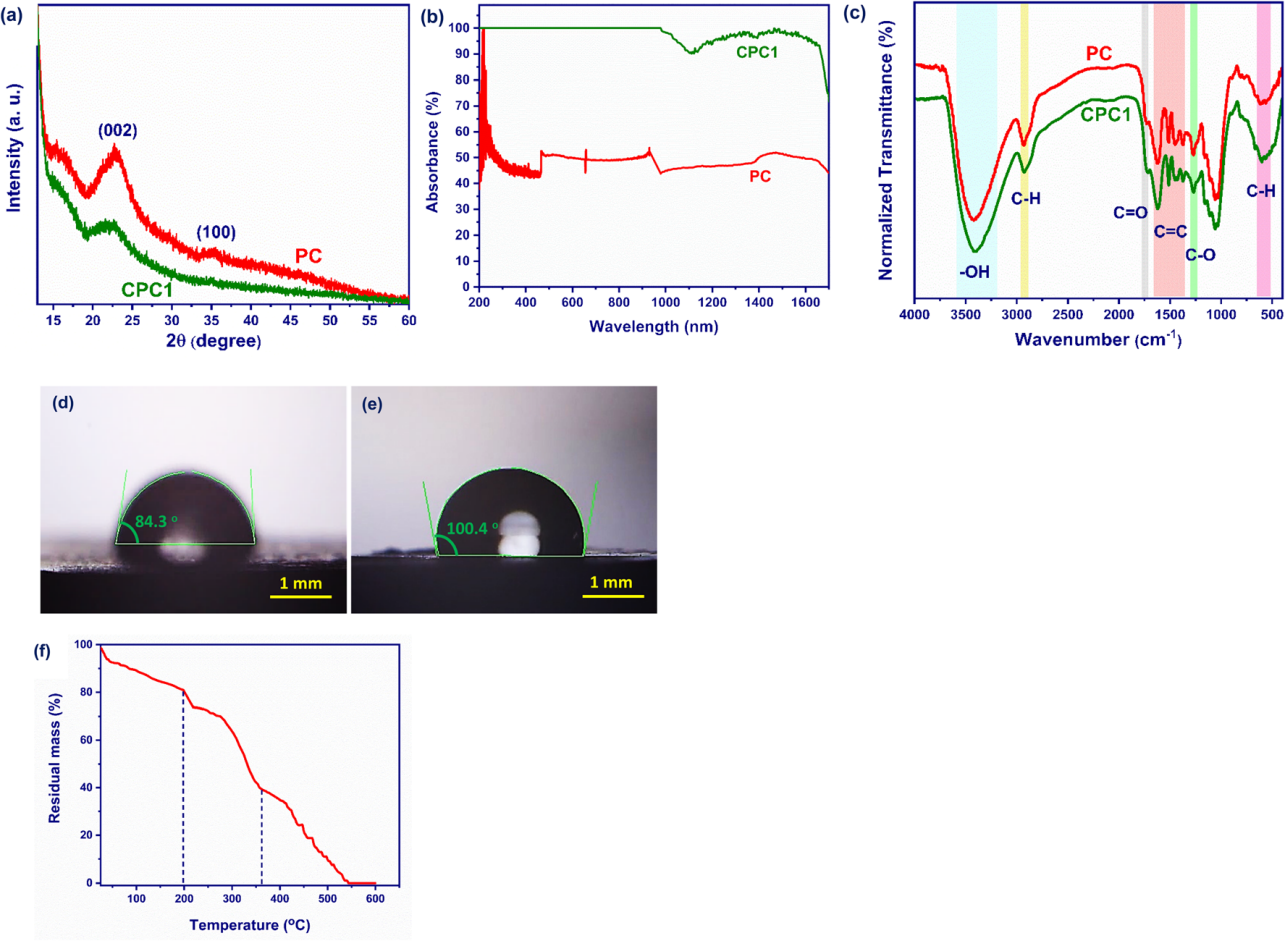
(a) XRD patterns, (b) absorption spectra, (c) FTIR spectra, (d) and (e) WCA of PC and CPC1. (f) TGA of CP.

Fig. 3a presents the XRD patterns for PC and CPC1. The characteristic peaks for PC at 22.9° and 35.3° correspond to the (002) and (100) diffraction planes, and represent the crystalline structure of the cellulose fibers in the cone scales.^59,65^ The position of the (002) peak of CPC1 was shifted to 22.5° with respect to PC, which was at 22.9°, but the peak intensity was decreased considerably. The reason for this reduction was due to the formation of amorphous carbon layers on the PC during the carbonization process.^65,66^ Also, the (100) peak of PC was completely removed after carbonization in the XRD pattern of CPC1.

Fig. 3b shows the absorption spectra of PC and CPC1 in the UV-Vis-NIR region in the wavelength range 200–1700 nm. The average light absorbances of PC and CPC1 were calculated as 48.699%. and 97.745%, respectively. After carbonization of the pine cone, its surface becomes black and rough, which leads to its increased light absorption in the UV-Vis-NIR region.

The FTIR spectra were used to identify the functional groups on the surfaces of PC and CPC1 (Fig. 3c). The bands at 3420 and 3401 cm^−1^ were attributed to the O–H stretching vibration mode of cellulose in PC and CPC1, respectively.^67^ The bands at 2931 and 2927 cm^−1^ of PC and CPC1 were assigned to the C–H stretching vibration of hemicellulose, respectively.^68^ The bands at 1723 and 1716 cm^−1^ were assigned to the C=O of hemicellulose in PC and CPC1, respectively.^69^ The bands at 1623, 1513, 1451 cm^−1^ in PC, and 1622, 1512, and 1450 cm^−1^ in CPC1 were due to C=C vibrations into the aromatic rings of the lignin biopolymer. The bands at 1270 and 1269 cm^−1^ originated from C–O bonds in PC and CPC1, respectively. The strong band at 600 cm^−1^ was related to C–H stretching vibration of the aromatic rings. The intensity of the C–H bond signals increased after the carbonization process.

The wettability behaviors of PC and CPC1 were measured by pouring 5 μL of DI water on their surface. As shown in Fig. 3d and e, the average water contact angles (WCAs) of PC and CPC1 were calculated as 84.3° and 100.4° at the initial time, respectively. Hence, CPC1 was slightly more hydrophobic than PC due to the presence of amorphous carbon layers from the carbonization process.

To find the carbonization temperature, the thermal degradation behavior of PC was investigated by TGA and the results are shown in Fig. 3f. For this purpose, 10 mg of PC was heated from room temperature to 600 °C at a heating rate of 20 °C min^−1^ under a nitrogen atmosphere as a carrier gas. As shown in Fig. 3f, PC as a raw material decomposed in three steps during the combustion process: an initial decomposition, main decomposition, and a final decomposition. Under 200 °C, physically adsorbed water, low molecular weight volatile compounds, and trapped gases were released completely in the initial decomposition,^70^ and the residual mass of PC decreased to 80%. The lignocellulosic materials started to degrade at 200 °C. The main and final decomposition occurred between 200 °C to 545 °C, and was due to losing chemisorbed water molecules and the decomposition of hemicellulose, cellulose, and lignin.^71,72^ Afterward, flue gases such as CO_2_, CH_4,_ and, H_2_O were released, which further reduced the mass of PC during the process.^72^ Moreover, the combustion process of PC ended at about 545 °C. The carbonization process occurred in the main decomposition stage at 200 °C to 360 °C.^73^ The final residual mass of PC was 40% at this stage. For this reason, 200 °C as the carbonization temperature was chosen.

**Fig. 4:**
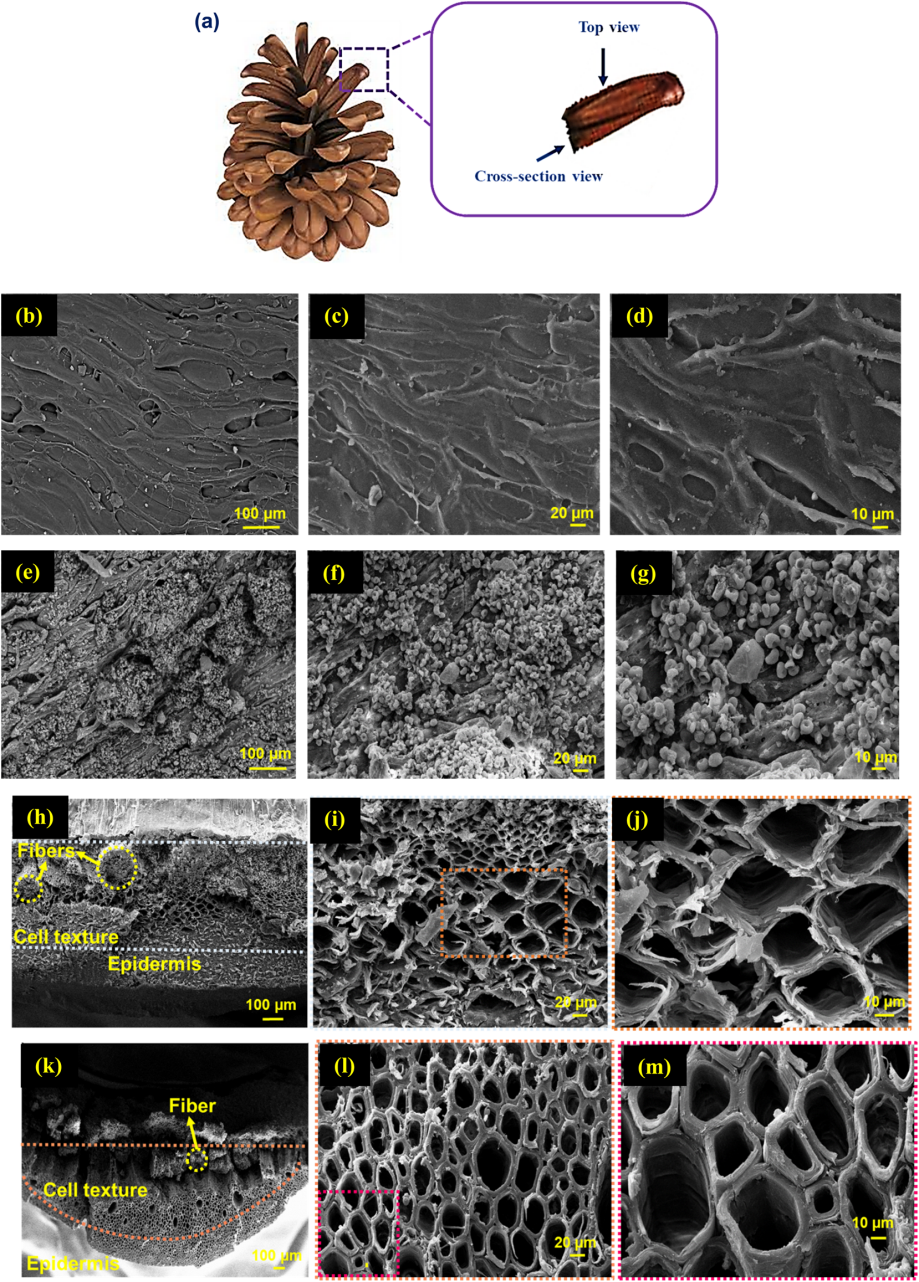
(a) Top and cross-sectional views. (b–d) SEM images of the surfaces of PC and (e–g) CPC1, and (h–j) cross-sectional views of PC and (k–m) CPC1.

The top surface and cross-sectional views of the PC scales are shown in Fig. 4a. As illustrated in Fig. 4b–m, the morphologies of the surface and channels of the PC scales before and after 1 h carbonization were probed through the obtained top (Fig. 4b–g) and cross-sectional views (Fig. 4h–m) from the SEM images. The morphology of the PC surface was uniform and smooth initially (Fig. 4b–d), while after 1 h carbonization, the surface of PC became rough and porous (Fig. 4e–g). The rough surface is a useful feature for light trapping and for enhancing the interfacial area for evaporation.^50^ As presented in the cross-sectional views (Fig. 4h and k), the cone scale was composed of an upper and lower part, named sclerenchyma fibers (cellulose fibers or fibers) and sclereids, respectively. All the layers of the cone scale take up water.^74^ The fibers boost the mechanical properties of the cone scales.^74^ The sclereids comprise the cell texture and epidermis,^75^ whereby the epidermis has a dense structure whereas the cell texture is porous, causing a gradient porosity in the lower parts of PC and CPC1, as depicted in the SEM images (Fig. 4h and k).^75^ The cell wall thickness, pore size, and shape of the interconnected porous structure of the cell texture of the PC scales change after 1 h carbonization (see Fig. 4i, j, l, and m). The average cell wall and pore size of CPC1 were measured as 5.3 and 26.09 μm, respectively, which were larger than those of PC (2.4 and 20.33 μm, respectively). The increase in the pore size of CPC1 in comparison with PC is beneficial for water transport and light trapping. In CPC1, the thickness of the cell wall increased and the shape of its pores became more regular with respect to PC due to its porous structure.

### 3.2. Evaporation performance of the photoabsorbers

**Fig. 5:**
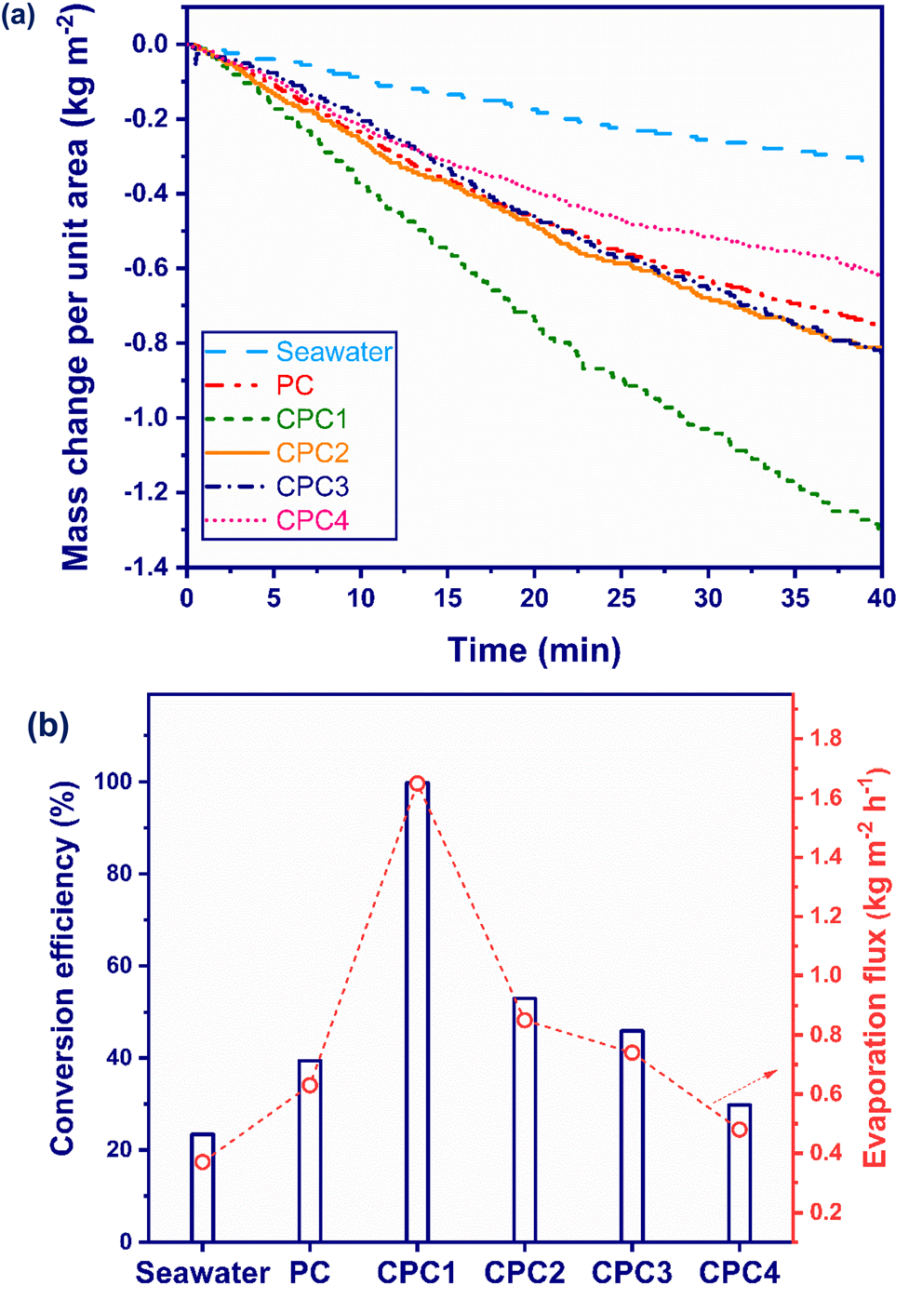
(a) Mass change per unit area and (b) photothermal conversion efficiency and evaporation flux of the seawater and the photoabsorbers under 1 sun solar illumination for 40 min.

The effect of the carbonization process time on the photothermal conversion efficiency of the photoabsorbers was investigated. Fig. 5a shows the seawater mass change per unit area *versus* the irradiation time for seawater, PC, CPC1, CPC2, CPC3, and CPC4 under 1 sun. The mass changes per unit area for seawater, PC, CPC1, CPC2, CPC3, and CPC4 were measured as 0.24, 0.42, 1.09, 0.56, 0.49, and 0.32 kg m^−2^, respectively, during 40 min under the simulated sunlight irradiation. Fig. 5b presents the conversion efficiency and evaporation flux of seawater and the photoabsorbers under 1 sun during 40 min illumination. The conversion efficiencies of seawater, PC, CPC1, CPC2, CPC3, and CPC4 were measured as 23.42%, 39.44%, 99.8%, 52.99%, 45.94%, and 29.82% during 40 min under 1 sun illumination. The minimum conversion efficiency was shown by seawater, which could absorb only 5% UV irradiation of the sunlight spectrum. The photothermal conversion efficiency of PC was less than that of the carbonized PCs (CPCs) except for CPC4. The brown color of the PCs was obviously converted to black after the carbonization process (Fig. S3†). Black materials can absorb more of the solar-light energy and generate heat on the water/air interface surface to improve the conversion efficiency of the ISSG process.^76^ Indeed, the oxygen functional groups of PC were broken and replaced with unsaturated C=C bonds during the carbonization process and hence their absorption of solar light increased in the NIR region to activate its orbital electrons.^77^ Among the photoabsorbers, CPC1, which was the optimum photoabsorber in this work, presented the maximum efficiency, with an evaporation flux about 4.5 times greater than that of seawater. The excellent evaporation performance of CPC1 as an ISSG device could be attributed to its high light-harvesting ability, enhanced interfacial area, high porosity, and rapid water transport. The conversion efficiency of the CPCs was affected by two main factors:

(1) With a decrease in the carbonization time, the radii of the CPCs were reduced under wet conditions in comparison with that under dry conditions, see Table S1.† In other words, the shrinking structures of CPC2, CPC3, and CPC4 did not change with respect to CPC1.

(2) With an increase in the carbonization time, the hydrophobicity of CPCs increased due to the formation of amorphous carbon layers on PC.

To determine the heat localization capability of CPC1, the Δ*T* of seawater was continuously recorded by the sensors at 40 and 60 min in light-on and -off conditions, respectively (Fig. S4†). As shown in Fig. S4,† Δ*T* rose under light-on condition for the top, medium, and bottom sensors. When the lamp was turned off, Δ*T* reached the equilibrium temperature and then decreased slowly, demonstrating the heat localization of the CPC1 surface.

**Fig. 6:**
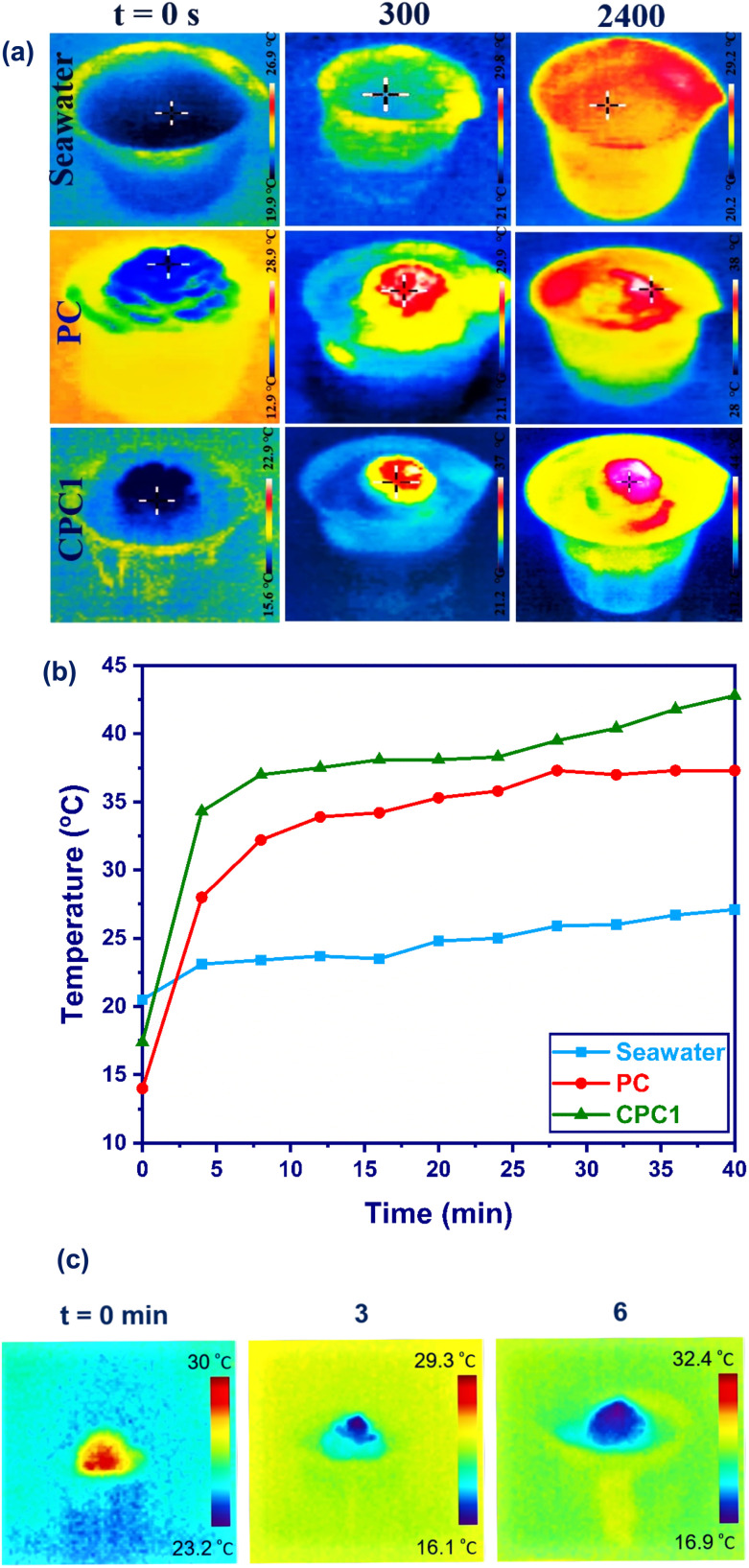
(a) Thermal images and (b) the surface temperature of the seawater, PC, and CPC1 from 0 to 40 min after contact with the water surface. (c) Thermal images of CPC1 respectively corresponding to *t* = 0, 3, and 6 min after contact with the water surface.

To record the temperature-gradient distributions of the seawater as a control and the photoabsorbers, an infrared (IR) thermal camera was used to capture photographs in the rainbow area. Fig. 6a shows the top surface temperatures of seawater, PC, and CPC1, which rose from 20.5 °C to 27.1 °C, 14.2 °C to 37.7 °C, and 17.5 °C to 42.8 °C during 2400 s exposure to 1 sun irradiation, respectively. As presented in Fig. 6a, after 40 min of irradiation, the temperature of seawater increased uniformly. No significant temperature increase was observed in the different parts except for the surface of CPC1. In other words, CPC1 could mainly reduce the heat loss to the bulk. That means, CPC1 could localize the produced heat at the interface by sunlight absorption while the solar irradiation was scattered in the bulk seawater. Fig. 6b shows the temperature changes at the surfaces of the seawater, PC, and CPC1 as a function of time under 1 sun. The surface temperature of CPC1 was measured as 11.9 °C and 6.6 °C greater than that of the seawater and PC during 5 min solar illumination. After 40 min, the temperature reached 27.1 °C, 37.7 °C, and 42.8 °C for the seawater, PC, and CPC1, respectively. The surface temperature differences for the seawater, PC, and CPC1 were measured as 6.6 °C, 23.5 °C, and 25.3 °C within 40 min under 1 sun, respectively. CPC1 showed the highest surface temperature difference.

The TC value of CPC1 in the dry and wet states was measured as 0.192 and 0.469 W m^−1^ K^−1^ at room temperature, respectively. The thermal insulation property of CPC1 was investigated by putting a fresh leaf on a hot plate, and CPC1 (height = 2.4 cm and diameter = 3 cm) (Fig. S5(a and b)†). The leaf on the hot plate was dehydrated (Fig. S5(c)†) but its color and shape on CPC1 did not change after 10 min heating at 180 °C (Fig. S5(d)†). Also, the top of CPC1 was cold. After heating, the IR image of CPC1 in Fig. S5(e)† indicated that the top temperature of CPC1 was 32.3 °C.

The water-absorbing capacity of CPC1 was investigated and is shown in Fig. 6c. The wetting process was rapidly completed within 6 min. Indeed, water was absorbed from the bottom (rachis) to the top surface of the cone scales of CPC1. The temperature of CPC1 was 28.1 °C before water absorbing. After water absorption for 3 and 6 min, the temperature of CPC1 was reduced to 19.5 °C and 18.6 °C, respectively. Hence, CPC1 had the ability to absorb and transport water to its surface, which is a necessary requirement for a photoabsorber in ISSG.

### 3.3. Heat-loss analysis

Thermal management by minimizing the heat-loss paths is an important feature of ISSG devices. The heat loss of CPC1 through conduction, convection, and radiation was calculated (Fig. S6†). The conduction heat loss of CPC1, *q*_cond_, can be obtained according to eqn (3):^19^3
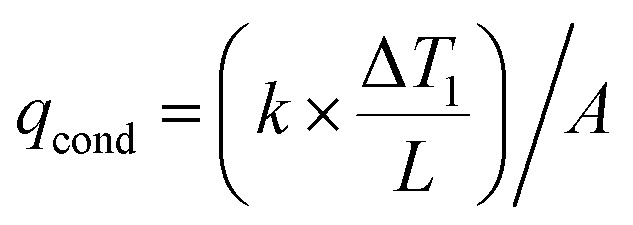
where *k* and 
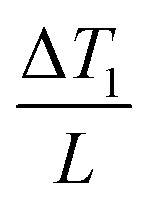
 are the TC of CPC1 in the wet state (0.469 W m^−1^ K^−1^) and the temperature gradient between CPC1 and ambient air after 40 min solar illumination, respectively, and *A* stands for the effective surface area of CPC1. The conduction heat flux of CPC1 was measured as 15.83 W m^−2^ or 1.58% under 1 sun illumination within 40 min.

The convection heat loss between CPC1 and ambient air, *q*_conv_, can be calculated by eqn (4):^19^

4 *q*_conv_ = *h* × (*T* − *T*_ambient_)

where *h* is the heat-transfer coefficient (5 W m^−2^ K^−1^), and *T* and *T*_ambient_ are the average surface temperature of CPC1 (37.5 °C) and ambient temperature (30.5 °C), respectively. The convection heat flux was measured as 35 W m^−2^ or 3.5% under 1 sun illumination.

The radiation heat loss, *q*_rad_, of CPC1 to ambient air can be calculated using eqn (5):^19^

5 *q*_rad_ = *εσ*(*T*^4^ − *T*^4^_ambient_)

where *ε*, *σ*, *T*, and *T*_ambient_ are the emittance of the surface of CPC1 (0 ≤ *ε* ≤ 1), the Stefan–Boltzmann constant (5.67 × 10^−8^ W m^−2^ K^−4^), the average surface temperature of CPC1, and the ambient temperature, respectively. The radiation heat flux was calculated as 46 W m^−2^ or 4.6% under 1 sun illumination. Hence, the high conversion efficiency of CPC1 was largely due to its 3D-structured top surface, which helped to reabsorb the lost refection energy as well as thermal radiation. Also, its large water/air interface speeded up the rate of steam escaping.

### 3.4. Quality of the desalinated water

#### 3.4.1. Seawater

**Fig. 7:**
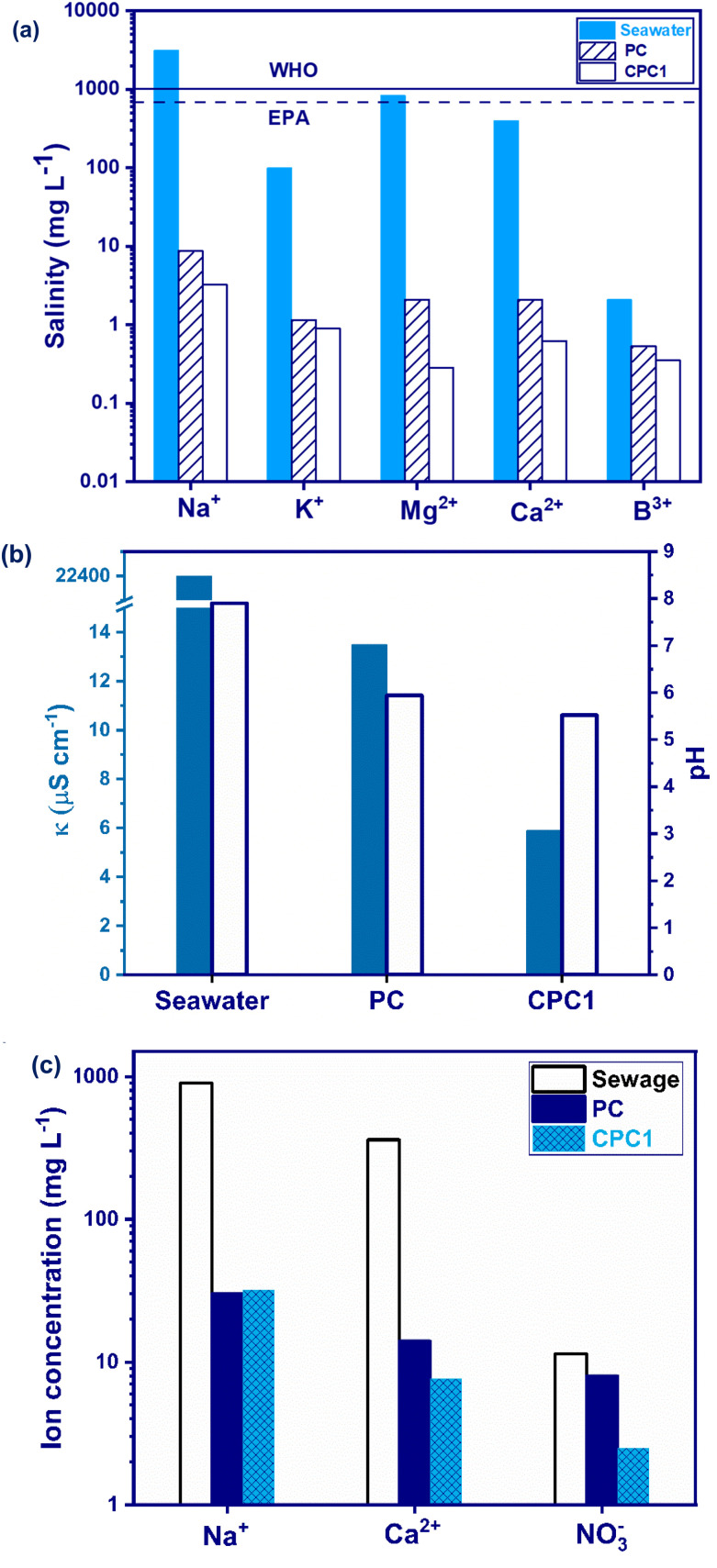
(a) Concentration of various ions, and (b) *κ* and pH of the seawater before and after desalination by PC and CPC1. The filled and unfilled bars represent the electrical conductivity and pH, respectively. (c) The ion concentrations of the sewage before and after purification by PC and CPC1.

The salinity of the seawater before and after desalination was measured by ICP-MS. As presented in Fig. 7a, the concentrations of all the ions after desalination by PC and CPC1 were much lower than the permissible values determined by the World Health Organization (WHO) and Environmental Protection Agency (EPA). The ion concentrations of Na^+^, K^+^, Mg^2+^, Ca^2+^, and B^3+^ in the desalinated seawater by PC and CPC1 were dramatically reduced. The salinity of the desalinated seawater by CPC1 was less than that of PC due to the greater evaporation flux of CPC1.

Considering the electrical conductivity, *κ*, and pH values are other ways to assess the quality of desalinated seawater. The *κ* of seawater was diminished from 22 400 to 13.5 and 5.9 μS cm^−1^ after desalination by PC and CPC1 (Fig. 7b). This shows that the concentration of ions had decreased considerably. Besides, the pH of the seawater decreased from 7.90 to 5.94 and 5.52 for PC and CPC1, respectively, due to the decrease in the alkaline and earth alkaline metal ions present in seawater.

#### 3.4.2. Wastewater

The industrial or agricultural discharge of several industries introduces several types of chemicals, like nitrite or nitrate ions, into the environment and pollute existing water sources.^78^ Nitrate ions represent a serious pollutant since they are very water soluble, and can cause eutrophication and several health complications, including methemoglobinemia and diabetes at high concentrations.^79,80^ The ability of CPC1 to remove nitrate ions in paper industry sewage as a wastewater source was investigated (Fig. 7c). CPC1 could not only desalinate seawater but also purify paper industry sewage. Fig. 7c shows that the concentrations of Na^+^, Ca^2+^, and NO^−^_3_ in wastewater decreased after purification by PC and CPC1. As Fig. 7c shows, both PC and CPC1 reduced the concentration of ions in the wastewater. The concentration of ions decreased more with CPC1 compared to PC due to its higher evaporation rate. This difference showed that CPC1 was much better for wastewater treatment because it harvested more sunlight than PC. Also, the pH of sewage changed from 7.7 to 6.9 and 6.6 after purification by PC and CPC1, respectively.

### 3.5. Cycling performance

**Fig. 8:**
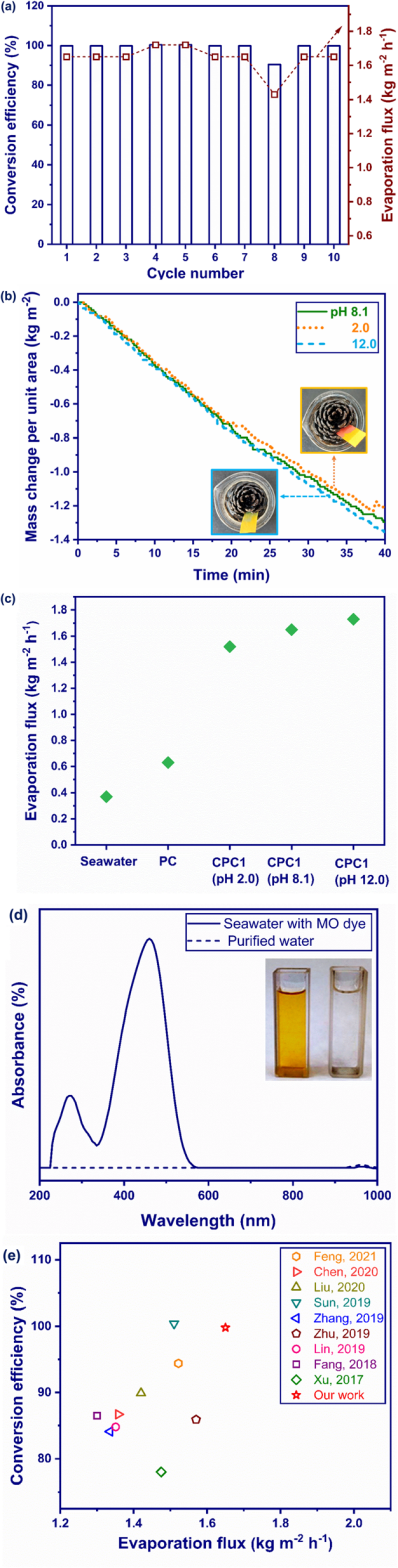
(a) Cycling performance of CPC1 at 1 sun in terms of the conversion efficiency and evaporation flux, (b) mass change per unit area for CPC1 at different pHs; insets correspond to the pH stability of CPC1: photographs after immersing in the acid solution (pH ∼ 2) and alkaline solution (pH ∼ 12). (c) Comparison of the evaporation flux of seawater, PC, and CPC1 under different pHs. (d) UV-Vis spectrum of seawater containing MO dye before and after desalination under 1 sun by CPC1. (e) Comparison of the conversion efficiency and evaporation flux of CPC1 with carbonized 3D photoabsorbers reported in the literature under 1 sun.

A photoabsorber should be sustainable and durable for practical applications. Salt fouling is the main challenge in ISSG, in which saturated salt ions are converted to crystals in the channels and pores of a photoabsorber,^81,82^ which can lead to a noticeable reduction in the evaporation flux and degradation of the photoabsorber.^83,84^ For this purpose, the performance of CPC1 as the best photoabsorber was continuously tested during 10 cycles under 1 sun illumination (Fig. 8a). The photothermal conversion efficiency and evaporation flux of CPC1 were found to be nearly stable during the 10 evaporation–condensation cycles. The carbonization of PC for 1 h not only increased its sunlight absorption, interfacial area, and water transportation through the porous structure of the pores to prevent the accumulation of salt crystals into its interconnected microchannels, but also decreases the heat loss.

### 3.6. Stability

The stability of CPC1 was tested under corrosive conditions in order to assess its use in practical applications. The evaporation performance of CPC1 at acidic (pH 2.0) and basic conditions (pH 12.0) was compared with that of seawater, PC, and CPC1 at normal seawater conditions (pH 8.1) (see Fig. 8b and c). As shown in Fig. 8b, no significant mass change was observed for CPC1 under acidic and basic conditions compared with under normal condition during 40 min under 1 sun, indicating the good stability of photoabsorber. The insets of Fig. 8b show photographs of CPC1 after immersing in acid and alkaline seawater. Besides, the evaporation flux values of seawater, PC, CPC1 (pH 2.0), CPC1 (pH 8.1), and CPC1 (pH 12.0) were measured as 0.37, 0.63, 1.52, 1.65, and 1.73 kg m^−2^ h^−1^, respectively during 40 min under 1 sun (Fig. 8c). Hence, CPC1 exhibited excellent evaporation performance in acidic and basic conditions.

### 3.7. Dye removal

In addition to desalination, the removal of pollutants like dyes in seawater is of great importance for providing freshwater. Organic dyes are extensively used in several industries, like textiles, printing, dyeing, electroplating, papermaking, and food processing.^85–87^ The anionic dye MO is one of the most common pollutants.^88^ In this work, 20 mg L^−1^ of MO dye solution in seawater was exposed to 1 sun illumination in the presence of CPC1. MO has the maximum absorbance at 508 nm.^89^ As the inset of Fig. 8d shows, the yellow color of MO solution in seawater became colorless in the presence of CPC1. Also, as Fig. 8d presents, the chromophore structure of the dye was removed. Hydrogen bonding was thus formed between the nitrogen and oxygen reactive groups of MO and the hydrogen atoms of hydroxyl groups in cellulose of CPC1 (Fig. S7†), and hence the dye was removed.^90^

### 3.8. Comparison with other photoabsorbers

Fig. 8e compares the conversion efficiency and evaporation flux of CPC1 with some 3D carbonized photoabsorbers reported in the literature under 1 sun, see Table S2† also. Fig. 8e confirmed that CPC1 had the best performance in terms of its conversion efficiency and evaporation flux among the other reported photoabsorbers, including bamboo,^56^ sunflower,^50^ and lotus seedpods.^52^ This is due to the appropriate features of CPC1, including its high solar-light absorption, low heat dissipation, and good water transportation through its porous structure.

## 4. Conclusion

In this work, the ability of carbonized pine cones as a highly efficient, low-cost, and scalable photoabsorber in ISSG and water purification was demonstrated. The porous structure of CPC1 could provide adequate water transport channels after carbonization. The unique structure of CPC1, such as its rough surface of cone scales, enhanced the water/air interface area for steam to escape and increased its absorption of sunlight. CPC1 had a high evaporation flux of 1.65 kg m^−2^ h^−1^ and conversion efficiency of 99.8% under 1 sun illumination. The concentration of various ions in seawater were reduced after evaporation–condensation in the presence of CPC1, such that the salinity of the desalinated water was much lower than that of the maximum safe values suggested by the WHO and EPA. The photothermal conversion efficiency and evaporation flux of CPC1 did not change significantly during 10 evaporation–condensation cycles. CPC1 exhibited good stability under corrosive conditions without significant change in its evaporation flux. Furthermore, CPC1 presented excellent performance in wastewater treatment. The chromophore structure of MO in the solution of MO dye in seawater was completely removed by CPC1 under 1 sun illumination. Also, CPC1 decreased the concentration of nitrate, sodium, and calcium ions in paper industry sewage. The pH of the sewage changed from 7.7 to 6.6 after purification by CPC1.

## Author contributions

Masoomeh Shafaee: investigation, validation, formal analysis, methodology, figures, tables, data curation, writing – original draft, visualization. Elaheh K. Goharshadi: supervision, resources, conceptualization, writing – review & editing, project administration, funding acquisition. Mohammad Mustafa Ghafurian: conceptualization, writing – review & editing. Mojtaba Mohammadi: methodology, writing – review & editing. Hassan Behnejad: supervision, funding acquisition.

## Conflicts of interest

There are no conflicts to declare.

## Supplementary Material

RA-013-D3RA01938A-s001
