# Supplementary material for: A highly efficient and sustainable photoabsorber in solar-driven seawater desalination and wastewater purification
Source: RSC Adv. 2023 Jun 14;13(26):17935–46. doi: 10.1039/d3ra01938a (PMC10265138; doi:10.1039/d3ra01938a)
Supplement: RA-013-D3RA01938A-s001 [file RA-013-D3RA01938A-s001.pdf]

Electronic Supplementary Material (ESI) for RSC Advances.
This journal is © The Royal Society of Chemistry 2023

Highly efficient and sustainable photoabsorber in solar-driven seawater desalination and wastewater purification

Masoomeh Shafaee, Elaheh K. Goharshadi, Mohammad Mustafa Ghafurian, Mojtaba Mohammadi, and Hassan Behnejad

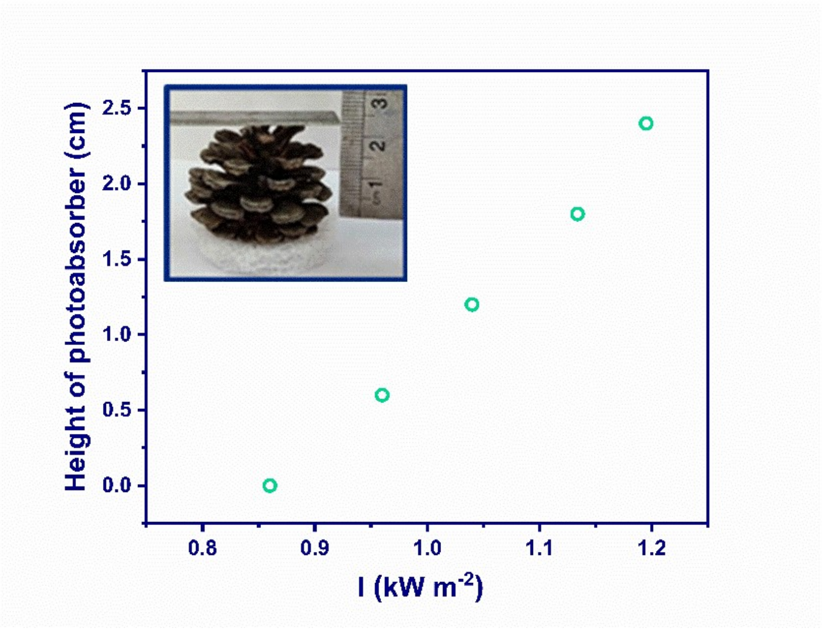

Fig. S1 The power density change of the solar simulator according to the height of CPC1.

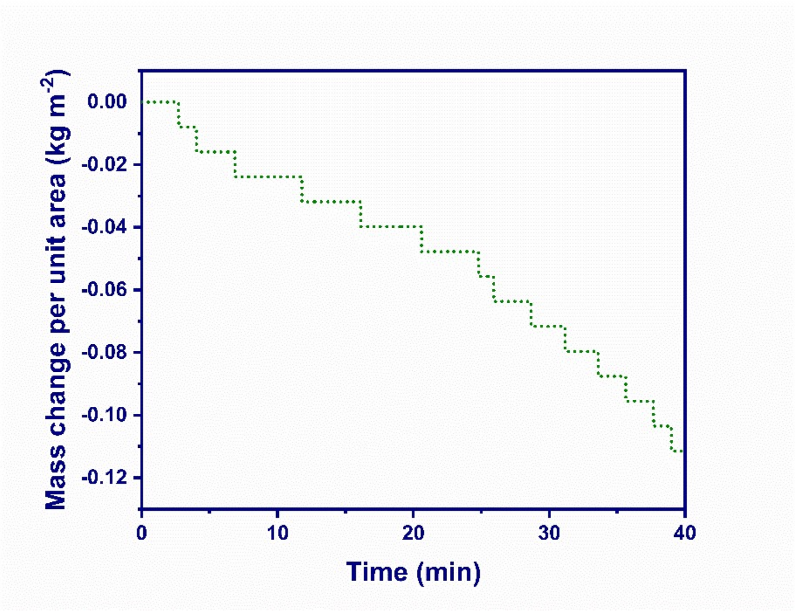

Fig. S2 The mass change per unit area of CPC1 under dark versus irradiation time.

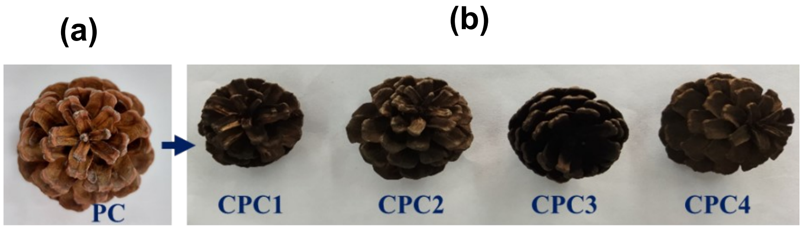

Fig. S3 The color change of (a) PC after (b) 1, 2, 3, and 4 h carbonization process.

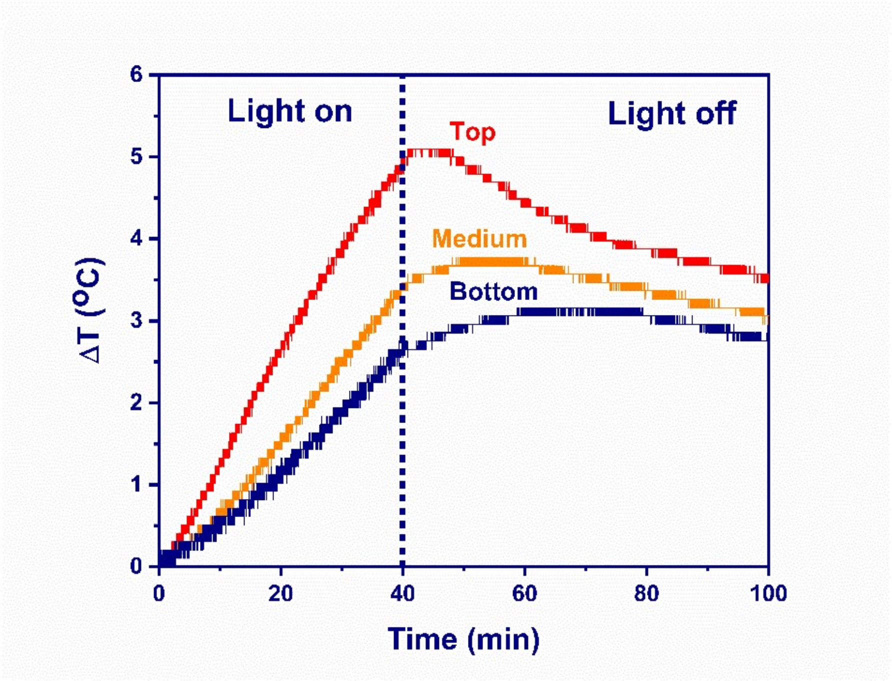

Fig. S4 Temperature change of three sensors of the bulk seawater in the presence of CPC1 under 1 sun for 40 min irradiation and 60 min dark.

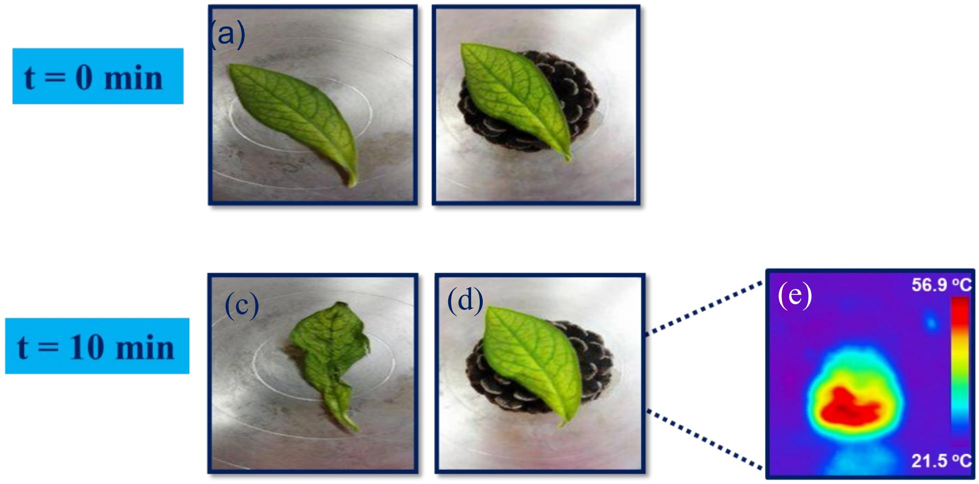

Fig. S The leaf on a hot plate and CPC1 (a and b) before (c and d) and after 10 min heating at 180 °C (e) IR image of CPC1 after heating.

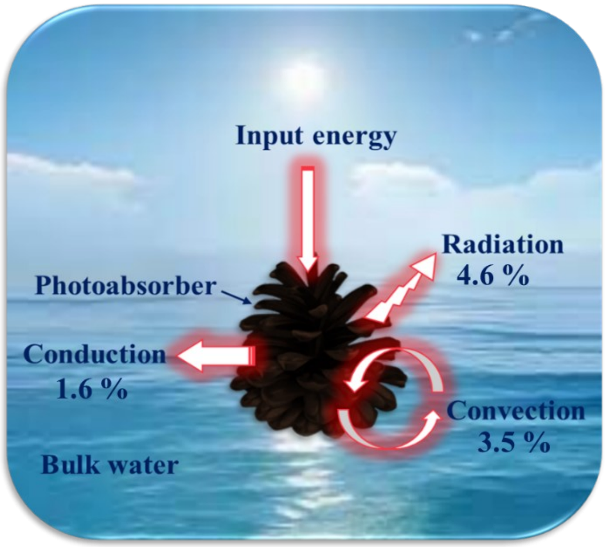

Fig. S6 Heat loss of CPC1 by conduction, convection, and radiation.

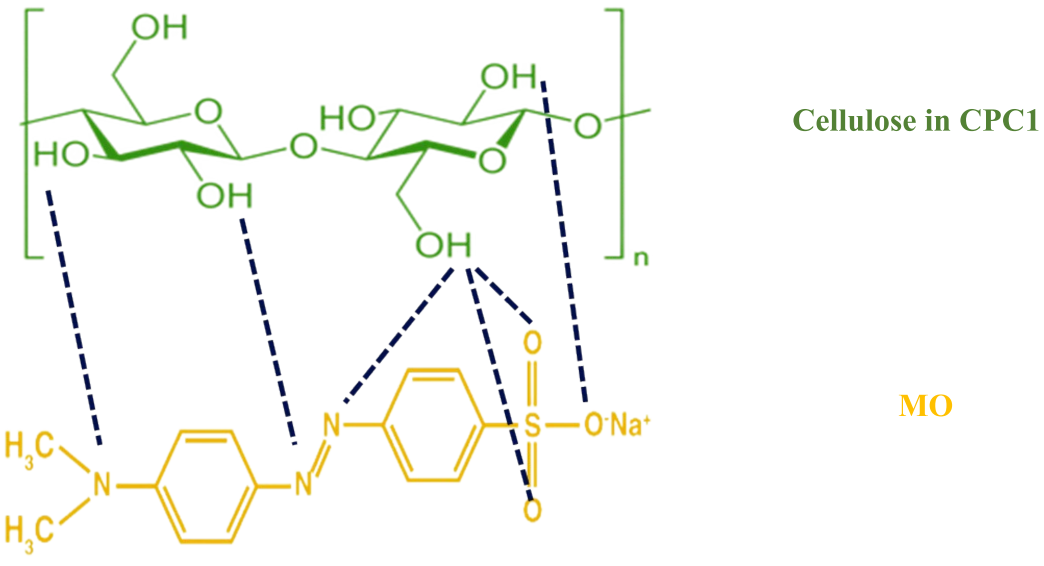

Fig. S7 Interaction between methyl orange (MO) dye and cellulose in CPC1.

Table S1 The required parameters of the seawater and the photoabsorbers for calculation of conversion efficiency and evaporation flux.

Sample	$\Delta r = \frac{r_{wet} - r_{dry}}{r_{dry}} \times$	Mass change per unit area (kg m^{-2})	Δm (g)	ΔT (°C)	ṁ (kg m^{-2} h^{-1})	% η
Seawater	-	0.24	0.31	5.65	0.37	23.42
PC	-30	0.42	0.77	2.36	0.63	39.44
CPC1	-28	1.09	0.95	3.43	1.65	99.8
CPC2	- 9	0.56	0.85	2.92	0.85	52.99
CPC3	0	0.49	0.57	3.13	0.74	45.94
CPC4	0	0.32	0.49	2.88	0.48	29.82

Table S2 Comparison of carbonized 3D photoabsorbers performances with CPC1.

Photoabsorber	Evaporation flux ($kg\ m^{-2}\ h^{-1}$)	Conversion efficiency (%)	Year
Bamboo	1.522	94.4	2021 [56]
Corncob	1.358	86.7	2020 [55]
Loofa	1.42	89.9	2020 [54]
Sunflower	1.51	100.4	2019 [50]
Pasta	1.3354	84.1	2019 [91]
Daikon	1.57	87.9	2019 [92]
Kelp	1.351	84.8	2019 [93]
Carrot	2.04	127.8	2019 [53]
Lotus seedpod	1.30	86.5	2018 [52]
Mushroom	0.198	78	2017 [49]
Pinecone	1.65	99.8	Our work
